# Electro-Spun Waste Polystyrene/Steel Slag Composite Membrane for Water Desalination: Modelling and Photothermal Activity Evaluation

**DOI:** 10.3390/membranes15100294

**Published:** 2025-09-28

**Authors:** Salma Tarek Ghaly, Usama Nour Eldemerdash, Ahmed H. El-Shazly

**Affiliations:** 1 Chemical and Petrochemicals Engineering Department, Egypt-Japan University of Science and Technology, New Borg El-Arab City 21934, Egypt; salma.ghaly@ejust.edu.eg (S.T.G.); usama.nour@ejust.edu.eg (U.N.E.); 2 Central Metallurgical Research and Development Institute (CMRDI), P.O. Box 87, Helwan 11722, Egypt; 3 Faculty of Engineering, Benha University, Beha 13511, Egypt; 4 Chemical Engineering Department, Faculty of Engineering, Alexandria University, Alexandria 21544, Egypt

**Keywords:** membrane distillation, electrospinning, waste polystyrene, steel slag, water desalination

## Abstract

Plastic waste and industrial residues like steel slag pose significant environmental challenges, with limited recycling solutions. This study investigates a sustainable approach by repurposing waste polystyrene and steel slag into composite membranes via electrospinning for membrane distillation applications. Steel slag incorporation enhanced membrane porosity, hydrophobicity, and thermal stability, with process optimization performed through response surface methodology by varying slag content (0–10 wt%), voltage (15–30 kV), and feed rate (0.18–10 mL·h^−1^). Optimized membranes achieved a reduced fiber diameter (1.172 µm), high porosity (82.3%), and superior hydrophobicity (contact angle 102.2°). Mechanical performance improved with a 12% increase in tensile strength and a threefold rise in liquid entry pressure over pure polystyrene membranes, indicating greater durability and wetting resistance. In direct contact membrane distillation, water flux improved by 15% while maintaining salt rejection above 98%. Under photothermal membrane distillation, evaporation rates rose by 69% and solar-to-thermal conversion efficiency by 60% compared to standard PVDF membranes. These results demonstrate the feasibility of transforming waste materials into high-performance, durable membranes, offering a scalable and eco-friendly solution for sustainable desalination.

## 1. Introduction

The problem of solid waste accumulation has reached alarming levels worldwide. Plastic waste, in particular, presents a persistent challenge because it does not break down naturally. Every year, nearly 300 million tons of plastic waste are generated globally, yet barely 9% gets recycled. The rest ends up in landfills or pollutes marine ecosystems. In the MENA region, waste production is notably high. Cities here see an average of 0.81 kg of waste per person each day, much of it plastic. Egypt stands out as one of the region’s largest contributors, producing roughly 5.4 million tons of plastic waste annually. Unfortunately, recycling systems are underdeveloped, leading to severe environmental consequences. The absence of efficient waste management in MENA countries, including Egypt, worsens pollution. There is a pressing need for better recycling initiatives and sustainable waste handling to address this growing crisis [1]. Polystyrene represents a significant portion of plastic waste globally, typically making up 10–20% of the total volume. Packaging remains one of its most common uses. In Egypt, where annual plastic waste reaches about 2.5 million tons, polystyrene numbers highlight its substantial environmental footprint alone, as it could add anywhere from 250,000 to 500,000 tons to that figure [2].

Globally, steel slag production is estimated to be around 300 million tons annually, representing about 10–15% of total steel output. In the MENA region, steel slag generation is significant due to the region’s robust steel production. In Egypt, steel slag waste is generated in substantial quantities, with estimates suggesting around 1 million tons are produced annually as the country aims to enhance its steel manufacturing capabilities. Effective management and recycling of steel slag remain critical challenges in the region and globally, as they hold potential for reuse in construction and other applications [3].

Water scarcity is a pressing global issue, with over 2 billion people currently experiencing water stress, primarily due to increasing demand, climate change, and pollution. The MENA region is among the most water-scarce areas in the world, with an average annual renewable water resource of just 1100 cubic meters per person, significantly below the global average of 6000 cubic meters. In Egypt, the situation is particularly critical, as the country relies heavily on the Nile River, which provides about 97% of its freshwater. With a population exceeding 100 million, Egypt’s per capita water availability has declined to approximately 600 cubic meters, nearing the threshold of absolute water scarcity. This alarming trend necessitates urgent action to improve water management and conservation strategies in both the MENA region and globally [4,5].

The incorporation of iron nanoparticles into membrane distillation (MD) systems has shown significant benefits for enhancing performance, as evidenced by recent research. Zhou et al. (2015) demonstrated that substituting Fe_3_O_4_ nanoparticles in graphene-based membranes improved structural integrity and hydrophobic properties [6]. Anvari et al. (2019) introduced a vacuum membrane distillation technique utilizing a self-heating membrane with dual hydrophilic-hydrophobic layers, enhancing energy efficiency [7]. Li et al. developed a Fe_3_O_4_/polyvinylidene fluoride-co-hexafluoropropylene (Fe_3_O_4_/PVDF-HFP) composite that achieved high permeate flux and salt rejection rates, highlighting the effectiveness of iron nanoparticles in solar-driven membrane distillation [8]. Furthermore, De León-Condés et al. (2019) reported that combining iron (II) oxide nanoparticles with sulfonated waste expanded polystyrene enhanced membrane performance and allowed for efficient oil/water separation [9]. These collective efforts underscore the significant potential of iron nanoparticles to improve MD performance and efficiency in desalination applications.

Electrospinning has emerged as a transformative technique for fabricating high-performance nanofibrous membranes for water treatment applications, offering unprecedented control over membrane morphology, porosity, and functional properties. This versatile manufacturing process utilizes electrostatic forces to draw polymer solutions or melts into continuous ultrafine fibers with diameters ranging from nanometers to micrometers, creating membranes with exceptional characteristics including high specific surface area (up to 1000 m^2^/g), interconnected pore structures, and porosity levels exceeding 80–90%. Recent advances in electrospinning technology have demonstrated remarkable achievements in membrane fabrication, with researchers successfully developing needleless electrospinning systems that significantly improve production rates from 1 to 5 mL·h^−1^ to 18 mL·h^−1^ and innovative coaxial electrospinning techniques enabling the creation of core–shell and hollow fibers with tailored functionalities. Current achievements in electrospun membrane applications for water treatment have shown exceptional performance metrics, including salt rejection rates exceeding 99.84% in membrane distillation systems, enhanced water flux improvements of up to 43% through optimized fiber morphologies, and superior antifouling properties with flux recovery ratios reaching 99.9% after multiple filtration cycles. The integration of advanced nanomaterials such as graphene oxide, metal–organic frameworks (MOFs), and inorganic nanoparticles into electrospun membranes has further enhanced their performance, with studies reporting water contact angles above 162° for superhydrophobic membranes and liquid entry pressures exceeding 186 kPa. Recent developments in green electrospinning using environmentally friendly solvents like Cyrene have addressed sustainability concerns while maintaining high membrane performance, while the successful electrospinning of waste polymers including expanded polystyrene has demonstrated both environmental benefits and effective water filtration capabilities with rejection efficiencies of 99.99%. These technological advancements position electrospinning as a leading manufacturing technique for next-generation membrane distillation systems, offering scalable, cost-effective, and environmentally sustainable solutions for addressing global water scarcity challenges [10,11,12,13,14,15,16,17,18].

Integrating photothermal materials into membrane distillation (MD) systems has shown significant promise for enhancing desalination performance by addressing temperature polarization, a key limitation of conventional MD. Unlike standard methods, photothermal materials enable localized solar heating directly at the membrane surface, raising surface temperatures and effectively reducing temperature polarization, which otherwise hampers vapor flux and efficiency. This localized heating approach minimizes preheating requirements and reduces energy consumption, making photothermal membrane distillation more efficient and scalable. Various materials, including noble metals, semiconductors, and carbon-based nanomaterials, efficiently convert light into heat, further improving MD’s thermal efficiency and making it a cost-effective solution for off-grid desalination in regions facing severe water scarcity.

This study addresses two pressing challenges: water scarcity and plastic waste accumulation, both globally significant and particularly acute in Egypt, where water shortages are severe and plastic waste, especially polystyrene, is substantial. The integration of membrane distillation (MD) technology, which effectively separates water from saline solutions, offers a dual solution by utilizing polystyrene waste in membrane fabrication. MD is advantageous due to its lower energy requirements and ability to operate on low-grade heat, making it more sustainable than traditional desalination methods. By repurposing waste materials in desalination, this approach enhances resource efficiency, contributing to both sustainable water management and waste reduction, and holds promising potential to alleviate water scarcity while tackling plastic pollution. In this context, electrospinning is employed as a versatile and efficient method for manufacturing MD membranes, particularly for incorporating waste polystyrene and steel slag as photothermal material. Electrospinning produces highly porous, nanofibrous membranes with improved water flux and selectivity, which outperforms conventional manufacturing techniques. Through Response Surface Methodology (RSM), the electrospinning process is optimized by adjusting variables such as steel slag dosage, voltage, and flow rate, yielding membranes with tailored characteristics. Photothermal analysis of these electrospun membranes further highlights their capability for effective light-to-heat conversion, enabling direct solar energy use in MD. This renewable energy integration positively impacts MD performance by addressing temperature polarization, lowering energy demands, and enhancing desalination efficiency, thus supporting both sustainable water treatment and waste repurposing efforts.

## 2. Materials and Methods

### 2.1. Materials and Chemicals

This study utilized transparent polystyrene (PS) obtained from discarded yogurt cups collected from local municipal waste sources. The collected plastic items were thoroughly cleaned, air-dried, and cut into small rectangular pieces measuring approximately 2 mm^2^ before further use. N, N-Dimethylformamide (DMF), procured from Fisher Chemicals (Waltham, MA, USA) with a purity of ≥99%, was used as the primary solvent in membrane fabrication. Distilled water was employed throughout all cleaning and solution preparation stages. For comparative membrane performance assessment, a commercially available poly(vinylidene difluoride) (PVDF) membrane sheet (Amersham Hybond P, Cytiva, Sweden, catalog number 10600057) was selected as a benchmark reference. Steel slag was sourced from Ezz Steel’s electric arc furnace (EAF) operations in Egypt. The slag was initially crushed using a mechanical mixer to reduce particle size and was subsequently ground to a final particle size of under 106 µm using a planetary ball mill (PM 400, MA Type, Retsch GmbH, Haan (Rhein-land, Nordrhein-Westfalen), Germany). Milling was performed with 200 g of slag and 117 g of grinding balls, in five 45 min runs.

### 2.2. Characterization

The functional groups and structural features of raw materials and finished membranes were examined using Fourier Transform Infrared Spectroscopy (FTIR; Bruker Vertex 70, Bruker Optics GmbH, Ettlingen, Germany), operating in the 4000–400 cm^−1^ range under ambient conditions. Crystalline structure and phase composition were determined via X-ray diffraction (XRD; Shimadzu XRD-6100, Kyoto, Japan). To analyze elemental composition, Energy-Dispersive X-ray Spectroscopy (EDX; EDAX APEX software 2.0) was used for polymer membranes, while X-ray fluorescence (XRF; Rigaku NEX CG EDXRF, Osaka, Japan) characterized the slag’s composition.

The average particle size of the steel slag was measured using a Zeta sizer (Malvern Nano Series, Malvern Panalytical, Herrenberg, Germany). Scanning Electron Microscopy (SEM; JCM-6000PLUS NeoScope, Tokyo, Japan) was utilized to examine membrane surface morphology [19]. Before imaging, samples were coated with a Pt/Pd alloy using a JEOL JEC-3000FC auto coater (40 mA, 3 Pa, 75 s). SEM images were analyzed with ImageJ software 1.53k to estimate mean pore sizes and distribution [20]. EDX mapping was also conducted to confirm slag integration into the membrane matrix [21].

Hydrophobic behavior was evaluated using static contact angle (CA) measurements with the sessile drop technique [22]. Membrane thickness was assessed using a Vernier caliper, with readings taken at five different spots and averaged.

Porosity was estimated via a gravimetric wet-dry method described in Section S2.1 [23,24]. The liquid entry pressure (LEP), crucial for ensuring hydrophobicity and operational stability, was determined using a custom dead-end filtration cell (Figure S1a). Mechanical strength was quantified using a universal testing machine (AG100KNXPLUS, SHIMADZU, Kyoto, Japan) operating at 100 kN with a loading rate of 5 mm·min^−1^, Figure S1b.

### 2.3. Waste Polystyrene/Slag Composite Membrane Preparation

To ensure high performance in membrane distillation (MD), the formation of beadless fibers is essential, as they enhance permeability, selectivity, and mechanical stability. In contrast, membranes exhibiting beaded fiber structures often suffer from enlarged pore sizes and structural inconsistencies, which diminish their effectiveness in MD operations. Consequently, average fiber diameter (AFD) was selected as the primary metric for assessing membrane quality, with smaller AFDs correlating to reduced pore sizes and improved separation capabilities—key attributes for efficient direct contact membrane distillation (DCMD) [25].

**Figure 1:**
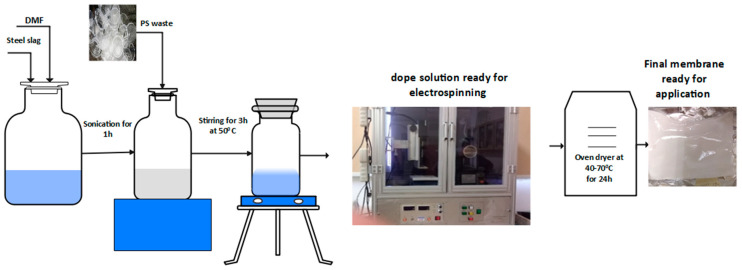
Membrane preparation experimental setup.

For membrane fabrication, varying concentrations of polymeric solutions (ranging from 10 to 35 wt%) were prepared in DMF solvent, as illustrated in Figure 1. These solutions were stirred at 50 °C for three hours to ensure homogeneity. In preparing the polystyrene-slag composites, steel slag was first ultrasonically dispersed in DMF prior to blending with the polymer solution, thereby promoting better dispersion within the final dope solution.

The electro-spinning setup used was NANON-01A (MECC CO., Ltd., Fukuoka, Japan). The electrospinning solution was loaded into a 5 mL plastic syringe connecting a # 21 steel needle. Positive voltage was regulated as needed. Different solution injection rates were tried for the composite membrane, and the electro-spinning time was adjusted to consume an equal solution. The tip-to-collector distance (TCD) was maintained at 15 cm., and the rotating drum collector was maintained at (100 rpm).

The plain transparent waste polystyrene was firstly electro-spun at different concentrations from 10 to 35 wt%) using the following spinning parameters: (0.5 mL·h^−1^, 100 rpm collector speed, 15 cm. tip to collector distance, and applied voltage of 27 kV) to obtain the polymer concentration that gives beadless fibers with small average fiber diameter (AFD). Alongside this, the effect of applied voltage on fiber diameter in electrospinning is a critical factor influencing the overall morphology and quality of the resulting fibers. A range of experiments was conducted at varying voltages between 17 and 27 kV. As a further matter, Optimal fiber formation is often achieved at lower feed rates as this permits adequate time for evaporation and facilitates the production of smooth, uniform fibers so that, the feed rate has doubled as this is highly desirable for time-saving [26]. Finally, the obtained optimized waste polystyrene membrane was then dried at 50 °C for 24 h and cold pressed at 0.654 MPa to enhance the membrane integrity.

### 2.4. Electrospinning Process Optimization Using Response Surface Methodology

The electrospinning of the polystyrene-slag composite membrane process was studied using the response surface methodology (RSM) to show the effect of the different parameters on the obtained fibers and further the membrane performance. BOX-BENKHEN design model was adopted as it proved its accuracy and adaptability with the electro-spun membranes where the number of runs were obtained as described in Section S2.2 [27]. The input parameters were selected to be slag dosage (0–10 wt% of soln.), applied voltage (15–30 kV), and the spinning rate (0.18–10 mL·h^−1^) at constant tip-to-collector distance (TCD) of 15 cm and rotating drum speed of 100 rpm and the fiber diameter was chosen as the main response to obtain the desired membrane characteristics.

### 2.5. Membrane Distillation Performance

#### 2.5.1. Direct Contact Membrane Distillation (DCMD) System Performance

For assessing the membrane performance, the optimized plain and composite membranes were tested for productivity using a customized membrane distillation system, and the PVDF commercial membrane was also tested for comparison. A hand-made direct contact membrane distillation (DCMD) unit was developed to test the performance of the obtained composite membrane using synthetic salt water with a salt concentration of 15,000 ppm. The membranes were tested continuously for 6 h, during which stable flux and salt rejection were observed, indicating initial operational stability. While longer-term testing is standard for comprehensive durability assessment, the 6 h stable performance aligns with common practices reported in the literature for preliminary MD evaluations [28]. The experimental flux was obtained, and the salt rejection was calculated using Equation (S13) [29].

The expected performance of the fabricated membranes was simulated using numerical modeling via Ansys software (2020 R2), employing the same 3D approach detailed by Rabie et al. A thorough description of the model is available in Section S2.3 [29,30,31,32,33,34,35,36]. The primary metrics used for evaluating system performance include the temperature polarization coefficient (TPC), membrane distillation thermal efficiency (η_MD_), and specific energy consumption (SEC) [30].

#### 2.5.2. Photothermal Activity Evaluation

The obtained membranes’ photothermal performance was evaluated using their temperature profile and water evaporation rate, which is estimated using the same system setup in Figure S4. The water evaporation rate experiments were carried out using a self-made photothermal evaporation system close to the one used by Xu et al. and fully described in Section S2.4 [37]. The mass change in the system with different membranes was recorded over 30 min with an electronic balance. The weight of the evaluating system is recorded, from which the evaporation rate (Em), kg·m^−2^·h^−1^ and the photothermal conversion efficiency (ɳsolar-thermal) is calculated using Equations (S21) and (S22) [37,38].

#### 2.5.3. Photothermal Membrane Distillation Evaluation

The photothermal membrane distillation (PMD) system performance was evaluated analytically and validated using literature data. Additionally, the commercial PVDF membrane to was tested experimentally using the same DCMD system setup, replacing the feed heater with a halogen lamp with intensity of 1 sun to show the effectiveness of using solar power on the resultant permeate flux. The analytical model adopted is based on the method described by Deoukchen Ghim et al. and fully depicted in Section S2.5 [31,36,39].

## 3. Results and Discussion

### 3.1. Raw Material Characterization

**Figure 2:**
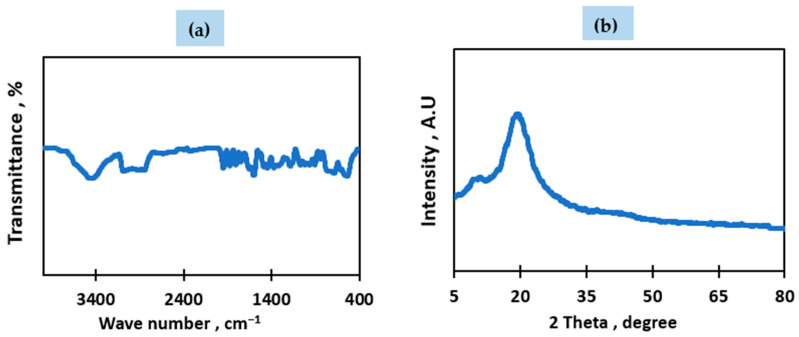
(**a**) FTIR of the waste transparent PS, (**b**) XRD of the waste transparent PS.

The structural properties and chemical composition of the waste materials and fabricated membranes were examined using several analytical techniques. The Fourier Transform Infrared Spectroscopy (FTIR) results for the waste transparent polystyrene, illustrated in Figure 2a, display multiple characteristic peaks. Peaks observed in the 538–696 cm^−1^ range are attributed to C–H bonds within aromatic rings [40]. A notable signal near 754 cm^−1^ corresponds to cis-1,4 polybutadiene, while peaks in the 964–979 cm^−1^ region relate to C–C torsion and in-plane bending of C–H, confirming the presence of butadiene structures [40]. Additional peaks at 748, 1600, and 1944 cm^−1^ further indicate aromatic monosubstituted benzene groups [41]. Peaks at 1447 and 1653 cm^−1^ are consistent with CH_2_ and C=C vibrations within the aromatic framework. The aliphatic C–H and CH_2_ asymmetric stretches are confirmed by peaks around 2922.6 cm^−1^, while aromatic C–H stretching appears at 3060 cm^−1^ [40].

Moreover, the presence of silicon-based additives is supported by bands at 1248, 1096, and 880 cm^−1^, corresponding to Si–CH_2_–R, Si–O–C, and Si–O stretching modes, respectively [42]. The broad peak near 3431 cm^−1^ indicates hydroxyl (–OH) functional groups [43,44].

X-ray diffraction (XRD) analysis, shown in Figure 2b, reveals broad diffraction peaks, suggesting that the transparent waste polystyrene possesses a semi-crystalline structure. The dominant peak at 2θ ≈ 20° confirms the presence of polystyrene as the main crystalline phase [45].

EDX analysis (Table 1) confirmed that carbon is the predominant element in waste PS, consistent with its organic nature. A minor silicon signal was also detected, supporting the presence of silicon-based additives indicated by FTIR. Although only carbon and silicon were quantified, it is worth noting that EDX is semi-quantitative, and other inorganic elements may have been present at trace levels below the detection threshold. These results establish a baseline composition for comparison with the PS/steel slag composite membranes.

**Figure 3:**
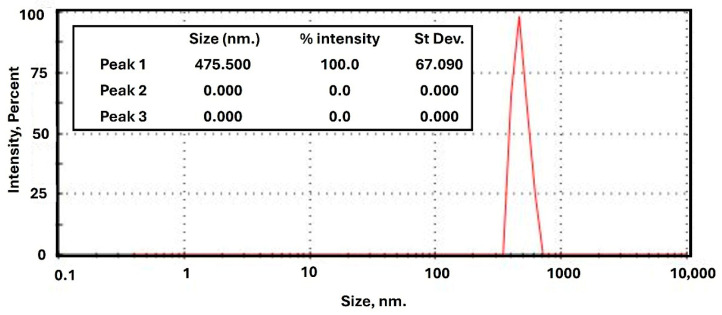
Zeta sizer analysis of the used steel slag.

Furthermore, the steel slag was characterized using X-ray fluorescence (XRF), and the results, as listed in Table 2, confirm that calcium oxide and iron oxide were the primary components, making it a suitable source of iron-based materials for composite membrane fabrication. Particle size distribution, determined by Zeta sizer analysis and shown in Figure 3, indicated an average particle size of 475.5 nm after grinding and processing.

### 3.2. Waste Polystyrene Electrospun Membrane Results

**Figure 4:**
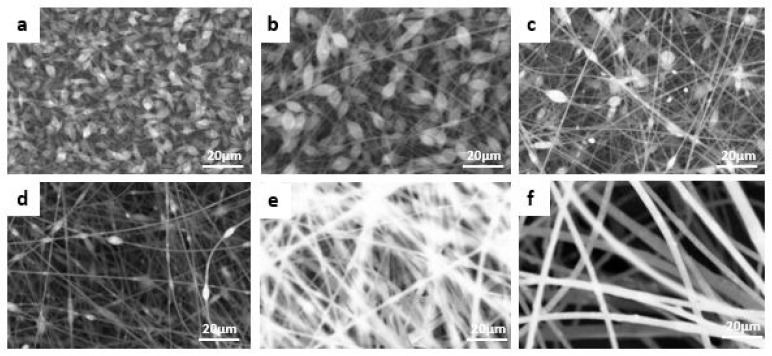
SEM morphology of fibrous transparent waste PS membranes at different polymer weight concentrations: (**a**) 10%, (**b**) 15%, (**c**) 18%, (**d**) 20%, (**e**) 25%, and (**f**) 35%.

Electrospinning experiments were conducted using transparent waste polystyrene at varying concentrations ranging from 10 to 35 wt%, as illustrated in Figure 4. The spinning conditions included a feed rate of 0.5 mL·h^−1^, a rotating drum speed of 100 rpm, a tip-to-collector distance of 15 cm, and an applied voltage of 27 kV. At lower polymer concentrations (10–20 wt%), a significant number of beaded fibers were observed. As the concentration increased to 25–35 wt%, the fibers appeared smoother and largely bead-free, with an average fiber diameter of approximately 4.60 ± 0.208 µm.

This trend can be attributed to the role of molecular chain entanglements in stabilizing the electrospun jet. At lower concentrations, insufficient chain entanglements lead to the formation of unstable jets, resulting in bead formation due to surface tension effects. Increasing the polymer concentration enhances solution viscosity and improves molecular entanglements, promoting uniform fiber formation. Additionally, with higher concentrations, bead shapes transitioned from spherical to spindle-like forms due to increased resistance to jet deformation [25].

**Figure 5:**
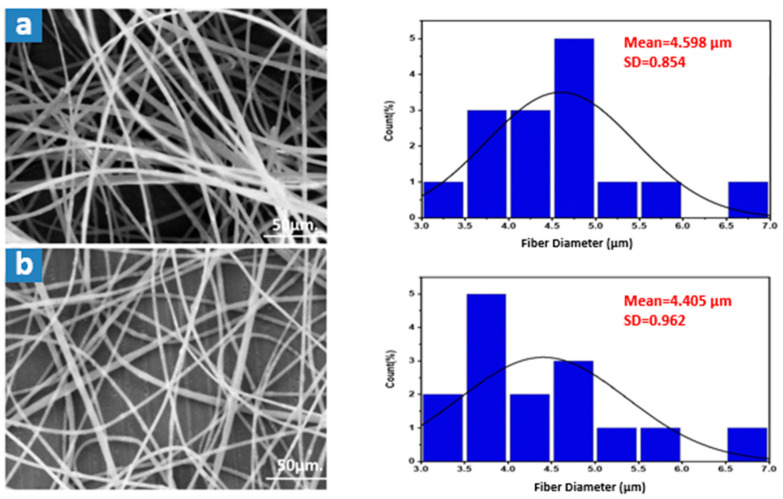
SEM morphology and the average fiber diameter (AFD) of transparent waste PS membranes at (**a**) 27 kV, and (**b**) 17 kV.

As shown in Figure 5, the effect of voltage on fiber morphology was also examined. A higher applied voltage led to a narrower fiber diameter distribution, whereas lower voltages (e.g., 17 kV) resulted in greater variability. The elevated voltage intensified electrostatic forces acting on the polymer solution, which typically facilitates fiber thinning. However, rapid solution ejection from the Taylor cone at high voltage can offset this effect and lead to slightly thicker fibers overall.

**Figure 6:**
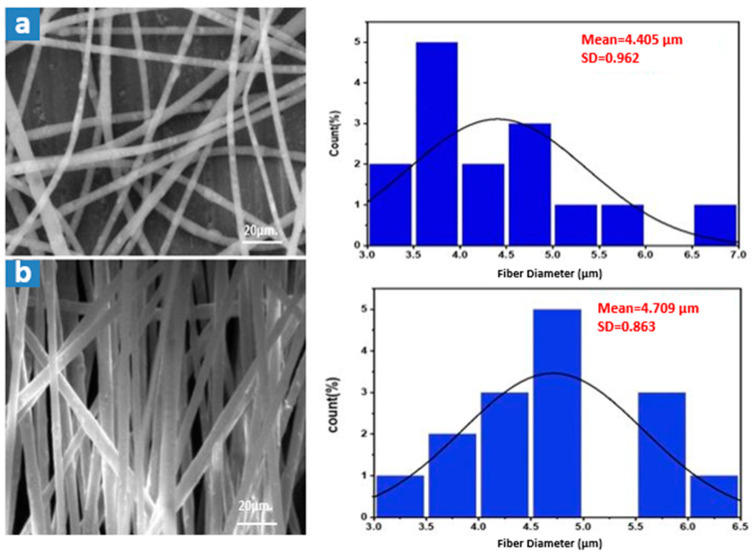
SEM morphology and the average fiber diameter (AFD) of transparent waste PS membranes at (**a**) 0.5 mL·h^−1^, and (**b**) 1 mL·h^−1^.

**Figure 7:**
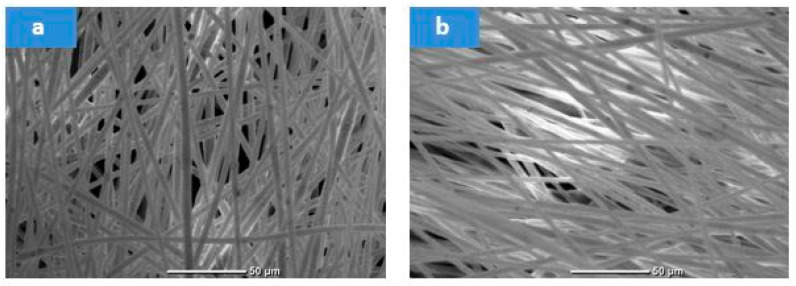
SEM morphology of the waste transparent polystyrene membranes at the optimum spinning conditions (17 kV, 1 mL·h^−1^, 15TCD, 100 rpm): (**a**) before pressing, and (**b**) after pressing.

The influence of the spinning feed rate is presented in Figure 6. Doubling the feed rate caused only a slight increase (~7%) in average fiber diameter. The denser fiber mats with minimal distribution spread observed at higher feed rates justified selecting the higher rate for subsequent trials. Following electrospinning, the membranes were dried at 50 °C for 24 h and cold-pressed, as illustrated in Figure 7. The final membranes displayed an increased average fiber diameter of 5.173 ± 0.097 µm.

### 3.3. Composite Waste Polystyrene/Slag Electrospinning Optimization Using RSM

The Box–Behnken model was utilized to obtain the model equation of the spinning process and optimize the process conditions to obtain the least uniform fiber diameter, resulting in minimum pore size and uniform distribution. Slag dosage, applied voltage, and spinning feed rate were manipulated as input model parameters, and the average fiber diameter was adapted as the response to the spinning process. The electrospinning parameters are set in Table 3, according to the range found in the literature, with coded levels of (−1 (minimum), 0 (center point), +1 (maximum)).

The suggested runs were performed, and the resulting fiber diameters were recorded, accompanied by the predicted and residual values according to the obtained responses (Table 4).

From ANOVA analysis, the Quadratic model showed the best fit with the highest R^2^ value and non-significant lack of fit, as shown in Table 5.

The model’s regression coefficients for the different parameters and their interactions suggested by the Quadratic model are presented in Table 6. The data show that the flow rate has the greatest effect on the obtained fiber diameter, followed by the filler dosage and the applied voltage.

From this, the coded quadratic equation could be driven to show the real effect of each independent variable on the obtained response as displayed:

Fiber diameter (Y) = 2.924 − 0.678 × A − 0.257 × B + 1.076 × C + 0.0189 × AB + 0.290 × AC − 0.057 × BC + 0.121 × A^2^ − 0.592 × B^2^ + 0.270 × C^2^

Moreover, the Actual model equation is derived from showing the actual values of the response with the actual values of the independent variables:

Average fiber diameter (µm) = −1.479 − (0.255 × Slag dosage(wt%)) +(0.445 × Applied Voltage (kV)) + (0.081 × Spinning rate(mL·h^−1^)) + (0.012 × Slag dosage (wt%) × Spinning rate (mL·h^−1^))–(0.002 × Applied Voltage (kV) × Spinning rate (mL·h^−1^)) +(0.005 × Slag dosage(wt%)^2^)–(0.011 × Applied voltage (kV)^2^) + (0.011 × Spinning rate (mL·h^−1^)^2^)

**Figure 8:**
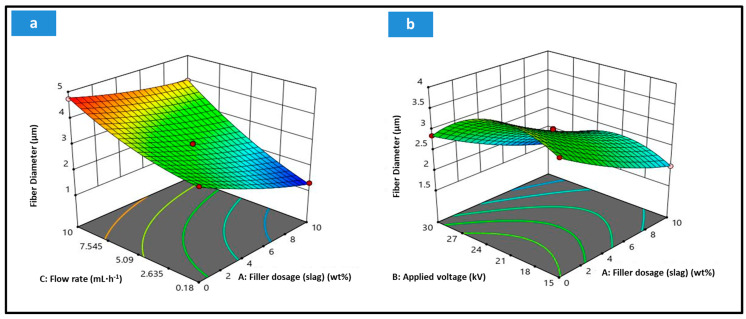
Three-dimensional surface response of the fiber diameter at center points with different input variables: (**a**) at Applied voltage (22.5 kV), (**b**) at spinning rate (5.09 mL·h^−1^).

A 3D surface response was obtained by keeping one of the variable constants at a zero level (center point) while varying the other two variables. The flow rate has the most significant effect, while the applied voltage has the least significant effect. At constant Applied voltage, Figure 8a, the fiber diameter slightly decreases with the increased slag dosage. Still, it vastly decreases with the decreasing spinning rate, reaching the lowest value at the maximum filler dosage and minimum spinning rate. As shown in Figure 8b, The fiber diameter decreases at center points with the increased filler dosage for the whole voltage range. However, the applied voltage has a controversial effect on the obtained fiber diameter. Below 22.5 kV, the fiber diameter increases with the applied voltage, reaching its maximum at the center point. Afterward, it is inversely proportional to the applied voltage from the center point to 30 kV. It hits its minimum value at the highest voltage with the maximum studied slag dosage.

The optimization of the chosen independent variables was performed with the aid of the software, setting the whole parameters in a range with medium importance, along with minimum fiber diameter with the highest importance. According to these goals, the software suggested 64 solutions to achieve the desirable aim. A number of these solutions were tested for model validation to show the model’s effectiveness in prediction, Table 7, and the optimum values were set to be as follows, Table 8, in line with desirability.

**Figure 9:**
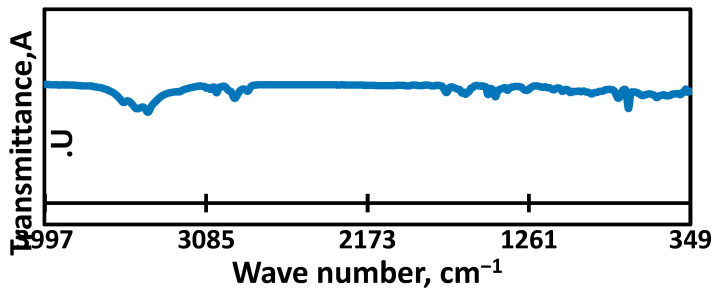
FTIR analysis of the optimized electrospun PS/steel slag composite membranes.

The obtained optimized membrane was fully characterized. The FTIR analysis of the obtained optimized composite membrane, as shown in Figure 9, reveals a series of characteristic peaks that correspond to various functional groups and molecular structures within the sample. The peaks in the range of 538–696 cm^−1^ are attributed to the C–H bond in the aromatic ring, with specific peaks at 754.61 and 695.93 cm^−1^ corresponding to CH out-of-plane bending vibrations of the phenyl ring. The aromatic C−H stretching is observed at 3025–3060 cm^−1^, while the aromatic monosubstituted benzene ring is indicated by peaks at 748, 690, 1049.2, 1600, 1748.2, and 1944 cm^−1^, with additional out-of-plane deformations at 756 cm^−1^ and 540–543 cm^−1^. Peaks at 1447 cm^−1^ and 1451–1653 cm^−1^ are assigned to the CH_2_ and C=C aromatic skeleton vibrations, respectively. The aliphatic C−H symmetric and asymmetric vibrations are observed at 3100–2800 cm^−1^, and asymmetric stretching and the bending vibrations of CH_2_ are seen at 1446.66 and 1491.99 cm^−1^, along with peaks at 2922.6 and 2921 cm^−1^. Polybutadiene is identified by a peak at approximately 754 cm^−1^ for cis-1,4 polybutadiene and peaks at 979−964 cm^−1^ corresponding to C−C torsion and C−H in-plane deformation. Silicon-containing groups exhibit distinct peaks: Si−CH_2_−R bond at 1248 cm^−1^, Si−O−C bond at 1096 cm^−1^, Si−O stretching modes at 880 cm^−1^, 950 cm^−1^, and 1000 cm^−1^, and Si−O asymmetric stretching, symmetric stretching, and bending vibrations at 1100 cm^−1^, 800 cm^−1^, and 460 cm^−1^, respectively. Lastly, the OH group shows peaks at 1638.1 cm^−1^ and 3400–3635 cm^−1^ for –OH bending vibrations, with a band at 3228 cm^−1^ indicating the stretching vibration of hydroxyl groups [43,44,52,53].

Iron oxides are indicated by strong peaks, particularly Fe_3_O_4_ at 580 cm^−1^ due to Fe–O vibrations from the magnetite lattice, with additional Fe–O stretching vibrations observed between 460 and 630 cm^−1^. The peak around 630 cm^−1^ is linked to Fe–O bonds from the ferrite skeleton, while the peak at 576 cm^−1^ corresponds to iron-occupied tetrahedral and octahedral positions, and the peak at 518.89 cm^−1^ indicates vibrations of Fe–O, Mg–O, Ti–O, and other metal oxides [6,47,54]. The Si–O–Al bond is reflected by a peak at 709 cm^−1^ [55]. CaCO_3_ is represented by a peak at 1420 cm^−1^ for doubly degenerate planar bending, 1093 cm^−1^ for symmetric stretching, and 880 cm^−1^ for out-of-plane and in-plane bending, along with a very weak vibration mode around 700 cm^−1^. Finally, a peak at 1620 cm^−1^ corresponds to the bending vibrations of adsorbed water on the iron oxide surface. A strong peak at 540 cm^−1^ is attributed to CaO [56], while the peak at 1487.49 cm^−1^ corresponds to C–O stretching vibrations, which are sometimes associated with increased CaO dosage [55].

**Figure 10:**
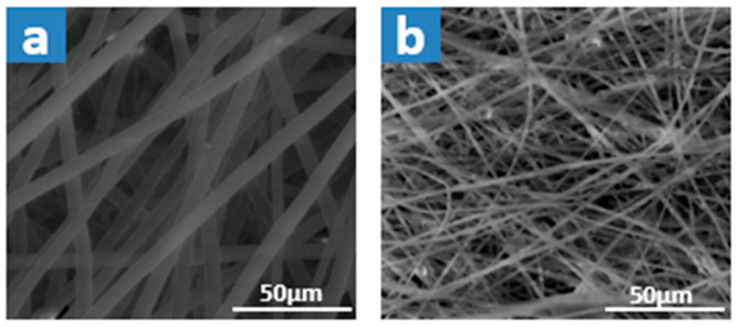
SEM morphology of 35% waste transparent PS membranes: (**a**) plain waste, (**b**) with optimized steel slag.

The SEM morphology of both optimized plain and PS/slag composite membranes, Figure 10, reveals that fiber diameter decreased from 5.173 ± 0.097 µm with plain waste to 1.172 ± 0.045 µm with optimized slag. In addition, the EDX analysis ensured the inherence of slag in the polymer matrix, as shown in Table 9, as the Fe element appeared in the obtained composite membrane.

As reported in our previous work [57], the incorporation of steel slag into the polymer solution reduces viscosity, which in turn decreases the likelihood of strong polymer aggregation and bead formation during electrospinning. In addition, increasing the solution’s conductivity due to the metal oxides present in the steel slag contrasts with the decrease in conductivity observed with higher polymer concentrations. Increased conductivity resulted in a higher net charge density on the electrospun jet. This higher charge density enhanced the elongation forces (electrostatic or Coulombic repulsion forces) on the PS solution during solvent evaporation and in the presence of an electric field, leading to greater elongation during electrospinning to obtain thinner fibers [25]. All these, as displayed in Table 10, prompt membranes with narrower pore size, increased porosity, and enhanced hydrophobicity.

The Liquid entry pressure and the tensile strength of the prepared membranes, Table 11, have been tested as the implications for long-term operation stability. The results reveal that adding steel slag increases the LEP, ensuring better wettability resistance. Moreover, the tensile strength of the obtained membranes improved, showing better mechanical tolerance and longer lifetimes for the obtained membranes.

### 3.4. Direct Contact Membrane Distillation Testing Results

**Figure 11:**
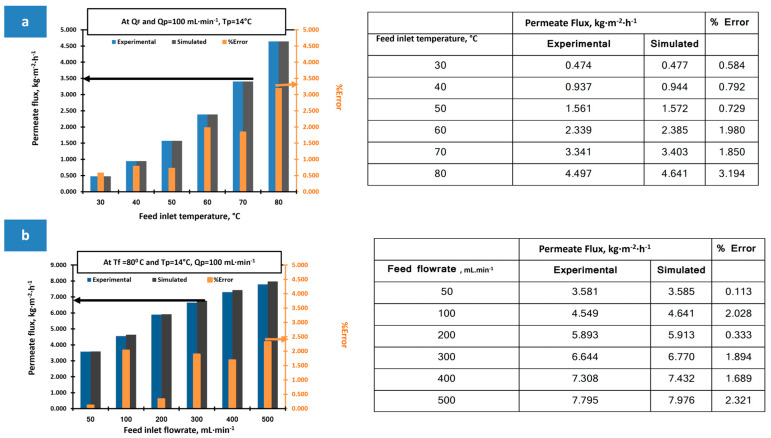
DCMD experiments using commercial PVDF membrane and Ansys model validation at different: (**a**) feed inlet temperatures, and (**b**) feed flow rates.

The membrane distillation (MD) model was verified using a commercial PVDF membrane across a range of feed inlet temperatures and flow rates, as shown in Figure 11a,b. The results indicated that system productivity improved with increases in both parameters. This enhancement is attributed to more efficient heat transfer within the feed channel at higher flow rates, which helps maintain the membrane surface temperature close to that of the feed bulk. This minimizes temperature polarization, resulting in a higher temperature polarization coefficient (TPC) and improved heat and mass transfer across the membrane, ultimately boosting the flux. As illustrated in the figures, the discrepancies between the simulated and experimental data remained within a 5% margin of error, confirming the model’s reliability for assessing the performance of the fabricated membranes.

**Figure 12:**
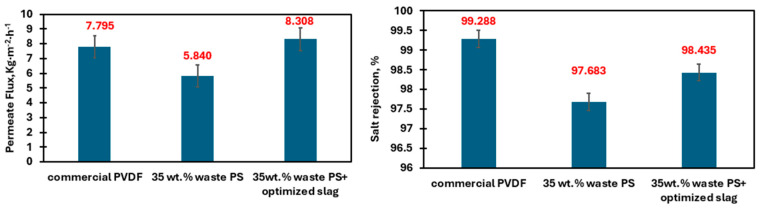
Experimental flux and salt rejection testing for the Commercial PVDF and the waste PS membranes.

The prepared membranes have been tested and simulated at 80 °C and 14 °C, at 500 and 100 mL·min^−1^, for the heated feed stream and permeate water temperatures and feed and permeate inlet flowrates, respectively. The main measured input MD characteristics for the different prepared membranes with the obtained flux are presented in Table 12. The experimental flux and salt rejection results as shown in Figure 12, showed an increase in the composite membranes compared to the PS membrane, which is attributed to the composite membrane’s higher porosity, which facilitates the passage of the vapor molecules through the membrane [19].

**Figure 13:**
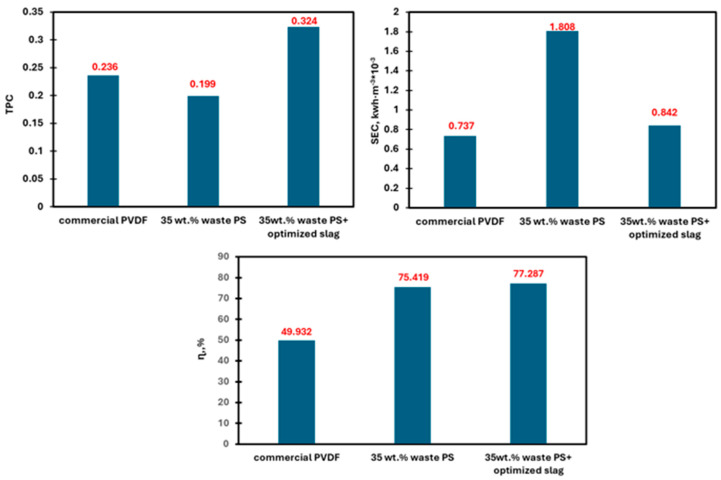
Performance numerical evaluation of the Commerical PVDF and the waste PS membrane.

The numerical testing showed an error of less than 5% with the experimental results in whole cases consequently, it is reliable to further evaluate the performance of the obtained membranes. As a result, it has been used to investigate the system efficiency as presented in Figure 13. The composite membranes displayed higher efficiency than the blank membrane, which could be explained by the increased membrane flux, which increases the amount of proper heat (Qv) used for water evaporation and increases the thermal efficiency according to Equation (S15). In addition, Figure 13 shows high TPC values for the composite membranes compared with the plain PS membranes, the reason may be due to the decreased thermal conductivity of the obtained membranes. Although silica and iron oxides are known for their higher thermal conductivity than PS, the resultant high porosity of the obtained membranes may counteract that and lead to decreased overall conductivity for the obtained membranes, according to Equation (S19). Hence, the membrane interface temperature could retain its value, leading to higher TPC values than plain PS membranes. Moreover, the enhanced properties of the composite membranes (porosity and pore size) result in an increasing mass transfer coefficient (C_m_), which leads to decreased SEC according to Equation (S20). Furthermore, the highly increased porosity improves the flux for the composite membrane. Consequently, it increases the heat utilized for evaporation Q_v_, Equation (S16), resulting in an improved thermal efficiency [21].

### 3.5. Photothermal Evaluation

#### 3.5.1. Membranes’ Temperature Profile

**Figure 14:**
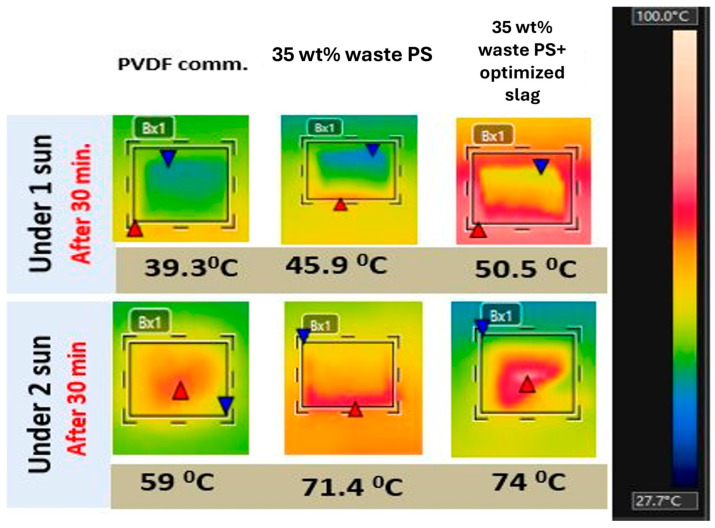
Surface temperatures of the different membranes under 1 and 2 suns.

The effective operation of a PMD system hinges on the photothermal active layer’s capacity to absorb solar energy efficiently. To see the materials’ response to solar illumination, one and two suns were illuminated for half an hour on the prepared membranes (2.5 × 2.5 cm^2^) and cooled for 3 min. IR camera photos were taken of the membranes’ surface, as presented in Figure 14. The whole membranes responded to solar illumination in less than 1 min. of illumination and the rate of temperature increase steadily decreases after 10min. of illumination. The effect of the whole fillers was positive on the temperature increase in the plain waste PS membrane. The prepared membrane surfaces showed the highest temperatures at the end of the heating period (30 min) of 50.5 for waste PS with slag, in comparison with 45.9 °C and 39.3 °C for waste PS and PVDF, respectively, and even higher under 2 sun to reach 59 °C, 71.4 °C, and 74 °C. The composite membrane showed the highest surface temperature as it contains several types of metal oxides with the ability of solar absorption. The steel slag used in this study mainly contains iron oxide (Fe_2_O_3_) and calcium oxide (CaO), as shown in XRF analysis (Table 2). The presence of Fe_2_O_3_ is significant for solar absorption as iron oxides have been extensively studied and utilized as solar absorber materials in various applications. While calcium oxides primarily contribute to the basicity and structural properties, these oxides can also have some influence on solar absorption due to their electronic properties [58,59,60].

#### 3.5.2. Evaporation Rate Experimental Results

In the photothermal system, the absorber layer plays a critical role by converting incoming solar radiation into thermal energy. This heat is subsequently transferred to the water transported via capillary action to the membrane’s surface, enabling sustained evaporation. The water supply to the evaporation site is maintained through interconnected porous pathways, ensuring continuous operation. Despite this, some portion of the generated heat is inevitably lost to the surroundings via conduction to the bulk water, radiation, and convection, which limits the overall evaporation efficiency. The porosity and thickness of the membrane directly affect light absorption and heat localization, thus influencing photothermal performance [37].

**Figure 15:**
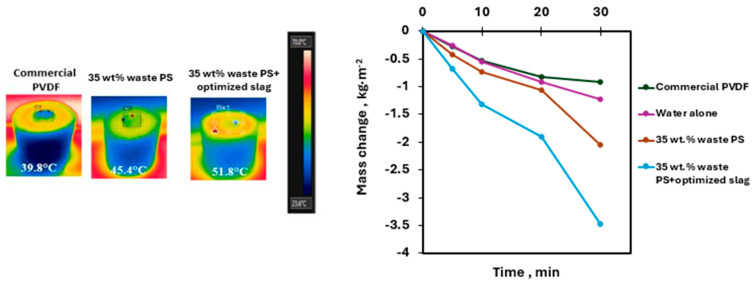
Evaporation rate surface temperature setup and the mass change results of the different tested membranes.

**Figure 16:**
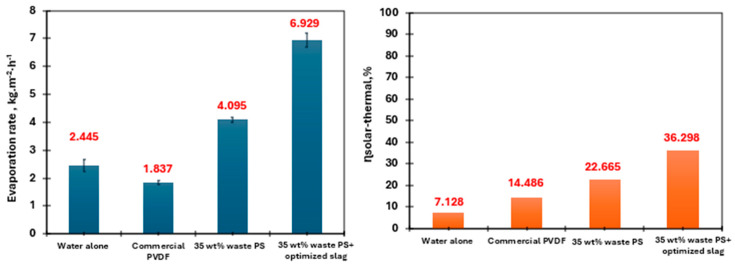
Evaporation rate and solar to thermal efficiencies of the different tested membranes.

According to the results shown in Figure 15 and Figure 16, after 30 min of solar exposure under 1-sun conditions, the membrane surface temperatures reached 39.8 °C for the commercial PVDF membrane, 45.4 °C for the waste polystyrene membrane, and 51.8 °C for the PS membrane enhanced with optimized slag. Correspondingly, the evaporation rates were recorded as 1.837, 4.095, and 6.929 kg·m^−2^·h^−1^, respectively. In contrast, the control sample (pure water) displayed a surface temperature of 25.0 °C with an evaporation rate of 2.445 kg·m^−2^·h^−1^. The relatively low evaporation rate of pure water can be attributed to its limited ability to absorb and utilize solar energy for heat generation. The enhanced evaporation observed in the composite membrane is primarily due to the improved photothermal effect provided by the added metal oxide fillers, which broaden the light absorption spectrum and increase solar energy conversion. Additionally, the use of thermally insulating foam beneath the membrane reduces heat dissipation, further supporting efficient evaporation. The solar-to-vapor conversion efficiency (η_th_) was determined using Equation (S22), with results shown in Figure 16, revealing a high photothermal efficiency of approximately 36%—a significant improvement compared to conventional materials, and well-aligned with advanced literature benchmarks [37].

#### 3.5.3. Photothermal Membrane Distillation (PMD) Results

The numerical model described in Section S2.5 is used with the following input parameters summarized in Table 13 as follows:

The obtained values for the whole output parameters for the commercial PVDF membrane are depicted in Table 14.

The obtained flux from the model has been validated using the PVDF commercial membrane and the literature data as depicted in Table 15.

As the revealed results show good agreement with the experimental results using PVDF and the literature values for DCMD with minor error % (less than 10%), the model has been used to predict the performance of the resultant PS and the composite membrane. The permeate flux values were obtained in Table 16, in the presence and absence of solar illumination.

The results reveal that using solar irradiation enhances the membrane flux by more than four times its value proving that the obtained composite membrane has higher photothermal conversion ability as previously assisted by solar evaporation rates and temperature profiles.

## 4. Conclusions

This study successfully demonstrates the potential of using waste polystyrene and steel slag to fabricate high-performance, eco-friendly nanofiber composite membranes optimized through Response Surface Methodology (RSM) for desalination. The optimized membrane achieved a minimized fiber diameter of 1.172 µm, increased porosity of 82.3%, and a high contact angle of 102.2°, yielding excellent hydrophobicity and a threefold improvement in wettability resistance (LEP) compared to virgin polystyrene membranes. These waste-derived composite membranes outperformed commercial PVDF membranes, showing a 69% increase in water evaporation rate and a 60% improvement in solar-to-thermal conversion efficiency. Notably, in Direct Contact Membrane Distillation (DCMD), the composite membranes provided a 15% increase in flux, while in Photothermal Membrane Distillation (PMD), water productivity improved fourfold, achieving salt rejection rates exceeding 98%. These findings highlight the viability of repurposing waste materials for sustainable water treatment solutions, contributing to a valuable approach toward reducing environmental waste and addressing water scarcity through innovative membrane technology.

## Figures and Tables

**Table 1:** EDX analysis of the used waste transparent PS.

Element, wt%	C	Si
	99.31	0.69

**Table 2:** XRF analysis of the steel slag.

Element, wt%	CaO	TiO_2_	Fe_2_O_3_	ZrO_2_	SiO_2_	Al_2_O_3_	MgO	MnO
	45.5000	0.4500	35.5000	-	9.0900	3.0300	2.0900	2.5200

**Table 3:** Independent variables and their corresponding levels for transparent waste polystyrene/steel slag spinning process.

Independent Variable	Symbol	Coded Levels			References
		−1	0	+1	
Slag Dosage, wt%	X_1_	0	5	10	[8,46,47]
Applied voltage, kV.	X_2_	15	22.5	30	[25,48,49]
Feed flow rate, mL·h^−1^	X_3_	0.18	5.09	10	[50,51]

**Table 4:** Experimental design runs of independent variables and the obtained experimental response.

	Independent Variables			Response		
Run Order	Slag Dosage(wt%)	Applied Voltage (kV)	Spinning Rate (mL·h^−1^)	Average Fiber Diameter (µm)		
				Actual Value	Predicted Value	Residual
**1**	5	22.5	5.09	2.96	2.92	0.0381
**2**	10	15	5.09	2.01	2.01	−0.0057
**3**	10	30	5.09	1.53	1.54	−0.0102
**4**	5	22.5	5.09	2.82	2.92	−0.105
**5**	5	22.5	5.09	2.91	2.92	−0.0164
**6**	5	15	0.18	1.69	1.73	−0.0344
**7**	5	30	0.18	1.3	1.33	−0.0298
**8**	5	30	10	3.4	3.36	0.0344
**9**	5	22.5	5.09	2.98	2.92	0.0611
**10**	10	22.5	0.18	1.31	1.27	0.0401
**11**	0	15	5.09	3.42	3.41	0.0102
**12**	5	15	10	4.02	3.99	0.0298
**13**	0	30	5.09	2.86	2.86	0.0057
**14**	10	22.5	10	3.98	4	−0.0241
**15**	0	22.5	10	4.74	4.78	−0.0401
**16**	5	22.5	5.09	2.95	2.92	0.0221
**17**	0	22.5	0.18	3.23	3.21	0.0241

**Table 5:** Design of experiment (DoE) suggested models.

Source	Lack of Fit *p*-Value	Adjusted R^2^	Predicted R^2^	
Linear	0.0008	0.8314	0.7171	
2FI	0.0005	0.8168	0.4275	
**Quadratic**	**0.6034**	**0.9962**	**0.9893**	**Suggested**
Cubic		0.9957		Aliased

**Table 6:** Regression coefficient values for fiber diameter of polystyrene/steel slag composite spinning process in terms of coded independent variables.

Factor	Coefficient Estimate
**Intercept**	2.92
**A-filler dosage (slag)**	−0.6779
**B-Applied voltage**	−0.2567
**C-Flow rate**	1.08
**AB**	0.0189
**AC**	0.2899
**BC**	−0.0571
**A^2^**	0.1208
**B^2^**	−0.5919
**C^2^**	0.2702
**R^2^**	0.9983

**Table 7:** Model validation using the suggested design of experiment (DoE) solutions.

Independent Variables				Response	

Slag Dosage (wt%)	Applied Voltage (kV)	Spinning Rate (mL·h^−1^)	Average Fiber Diameter (µm)		
			Actual Value	Predicted Value	Residual
10	29.189	0.18	0.65	0.639	0.011
10	21	0.18	1.492	1.284	0.208
10	15	0.429	0.935	0.905	0.03

**Table 8:** Optimum solution actual and predicted values.

Optimum Conditions	Coded Levels	Actual Levels	
**Slag dosage, (wt%)**	+1	10	
**Applied voltage, (kV)**	0	15	
**Spinning rate, (mL·h^−1^)**	−0.786	1.229	
**Response**	**Experimental values**	**Predicted values**	**Residual**
**Fiber diameter, (µm)**	1.172	1.061	0.111

**Table 9:** EDX analysis of the optimized electrospun PS/steel slag composite membranes.

%Element	C	Si	Ca	Ti	Fe	Zr	Mg	Al	Na	S
**Waste PS**	96.302	1.202	0.392	0.000	0.629	1.475	0.000	0.000	0.000	0.000
**Waste PS+ optimized Slag**	60.208	5.210	18.071	0.000	13.568	0.000	1.489	1.454	0.000	0.000

**Table 10:** Characteristics of transparent waste PS membranes and optimized steel slag ones.

Type	35wt% Waste PS Membrane	35wt% waste PS/Optimized Slag Composite Membrane
**Average pore diameter, µm.**	2.562 ± 0.225	0.736 ± 0.107
**Porosity, %**	71.764	82.331
**Contact angle, °**	99.448	102.179

**Table 11:** LEP and tensile strength of the different electrospun transparent waste PS membranes.

Membrane Type	Thickness, µm	LEP, bar	Tensile Strength, MPa
**Transparent waste PS**	320.00	0.12	1.020
**PS +optimized slag**	384.00	0.38	1.144

**Table 12:** Different obtained transparent waste membranes’ characteristics’ and their experimental and numerical performance.

Membrane Type	Porosity, %	Pore Size, µm	Thickness, µm	Flux (kg·m^−2^·h^−1^)			
				Experimental	Simulated	Error, %	Salt Rejection, %
**Waste PS**	72	1.281	320	5.840	5.867	0.460	97.683
**Waste PS + Optimized slag**	82	0.368	384	8.308	8.328	0.240	98.435

**Table 13:** The numerical model input parameters.

T_f_ = 29 °C, Tꝏ = 21 °C,	Qf = 500 mL·min^−1^,	S = 15,	**ɛ =** 0.95
	A = 0.062 × 0.062 m^2^		
	L = 0.0155 m		

**Table 14:** Obtained output parameters of the numerical model for the commercial PVDF membrane.

**Feed bulk parameters**		**ρ_w_**(kg·m^−3^) = 1017.665692, **ρ_s_** (kg·m^−3^) = 1028.830059	
		**β**(°C^−1^) = 0.001152551, **µ**(kg·m^−1^·s^−1^) = 0.001234 **∆L_vap_**.(KJ kg^−1^) = 2432.708064	
		**K**(W·m^−1^·°C^−1^) = 0.619434, **C_p_**(J·kg^−1^ ·°C^−1^) = 4103.956233	
		**R_e_** = 186.942384, **Pr** = 8.174362, **Nu** = 8.214267	
**Heat flux (W**·**m^−2^) values** **using initial value of (T_mf_ = T_f_ = 29 °C)**			
**Q_e_**	**Q_R_**	**Q_conv-feed_**	**Q_conv-membrane_**
111.840160	45.694920	2626.165073	2783.700154
**Tmf_0pt_** (°C) = 34.079747			

**Table 15:** Model validation using experimental permeate flux values of the commercial PVDF and other membrane types.

	Flux (kg·m^−2^·h^−1^)		Reference
	Experimental	Simulated	
**PVDF**	2.77	2.960	**This Study**
**JPTM**	1.290	1.421	[61]
**PAN-CB/PVDF/PP**	0.430	0.450	[62]
**PSP-SPH**	44.400	45.104	[63]

**Table 16:** Numerical flux values of the obtained electrospun PS composite membranes with and without solar illumination.

Type	T_mf_	PMD Flux, kg·m^−2^·h^−1^	DCMD Flux, kg·m^−2^·h^−1^
**Commercial PVDF**	34.080	2.960	0.607
**35 wt% waste PS**	34.285	3.159	0.474
**35 wt% waste PS+** **optimized slag**	34.542	3.181	0.700

## Data Availability

The original contributions presented in this study are included in the article and supplementary material. Further inquiries can be directed to the corresponding author(s).

## Supplementary Materials

The following supporting information can be downloaded at: https://www.mdpi.com/article/10.3390/membranes15100294/s1, Figure S1: (a) Dead-end filtration cell for LEP testing, (b) Universal testing machine for tensile; Figure S2: DCMD experimental setup: (1) Chiller, (2) Permeate container, (3) Permeate pump, (4) MD cell, (5) Thermocouples, (6) Data logger, (7) Feed pump, (8) Feed heater, (9) Feed container; Figure S3: Schematic diagram of the 3D model flow directions; Figure S4: Photothermal evaluation experimental system set-up; Figure S5: The experimental setup for water evaporation rate testing; Figure S6: Heat fluxes of the adopted photothermal membrane distillation model; Figure S7: Flow chart of the numerical model; Section S2: Supplemental calculations (S2.1. Porosity calculation; S2.2. Response surface calculations; S2.3. Numerical model of direct contact membrane distillation; S2.4. Photothermal Activity evaluation; S2.5. Photothermal membrane distillation evaluation; S2.6. Uncertainty Measurements).
