# Supplementary material for: Electro-Spun Waste Polystyrene/Steel Slag Composite Membrane for Water Desalination: Modelling and Photothermal Activity Evaluation
Source: Membranes (Basel). 2025 Sep 28;15(10):294. doi: 10.3390/membranes15100294 (PMC12566489; doi:10.3390/membranes15100294)
Supplement: Supplementary file 1 [file membranes-15-00294-s001.zip › membranes-3854427-supplementary.pdf]

Supporting Information

membranes

Electro-spun Waste Polystyrene/Steel Slag Composite Membrane for Water Desalination: Modelling and Photothermal Activity Evaluation

Salma Tarek Ghaly [1,2] **, Usama Nour Eldemerdash*[1,3]*, and A. H. El-Shazly*[1,4]

[1]Chemical and Petrochemical Engineering Department, Egypt-Japan University of Science and Technology, New Borg AL Arab city, Alexandria, Egypt.
[2]Central Metallurgical Research and Development Institute (CMRDI), PO Box 87 Helwan, Cairo, Egypt.
[3]Benha Faculty of Engineering, Benha University, Qaliobiya, Egypt.
[4]Chemical Engineering Department, Faculty of Engineering, Alexandria University, Alexandria, Egypt.

Keywords: Membrane distillation; Electrospinning; Waste polystyrene; Steel slag; Water desalination

Section 1 Supplemental Figures

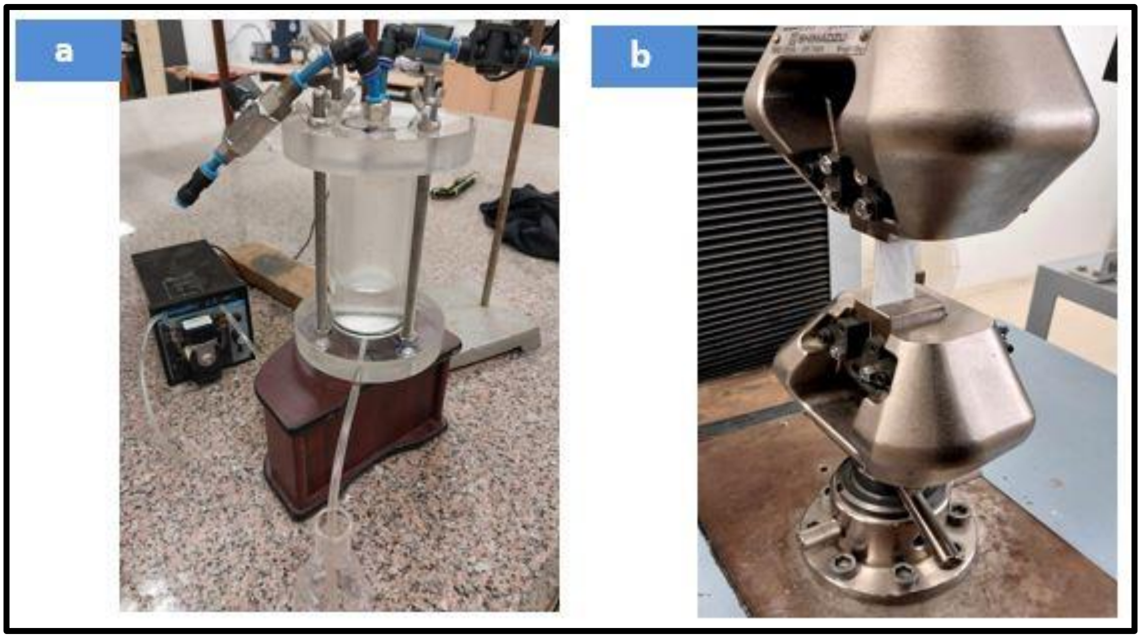

Figure S1. **(a)** Dead-end filtration cell for LEP testing, **(b)** Universal testing machine for tensile

- The experimental arrangement, as depicted in **Fig. (S.1.2).** included a DCMD cell (constructed from acrylic with dimensions of 6x6 cm^2, with feed and permeate channel heights of 12 and 6 mm., respectively), feed and permeate pumps, a chiller, a water bath (for heating the feed water), thermocouples, a data logger, and associated connections.

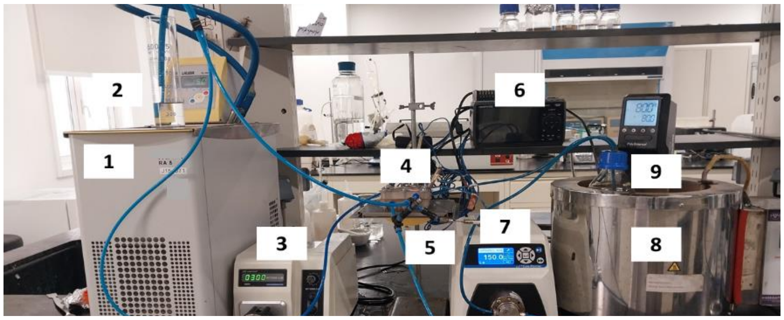

Figure S2. DCMD experimental setup: (**1**) Chiller, (**2**) Permeate container, (**3**) Permeate pump, (4) MD cell, (**5**) Thermocouples, (**6**) Data logger, (**7**) Feed pump, (**8**) Feed heater, (**9**) Feed container

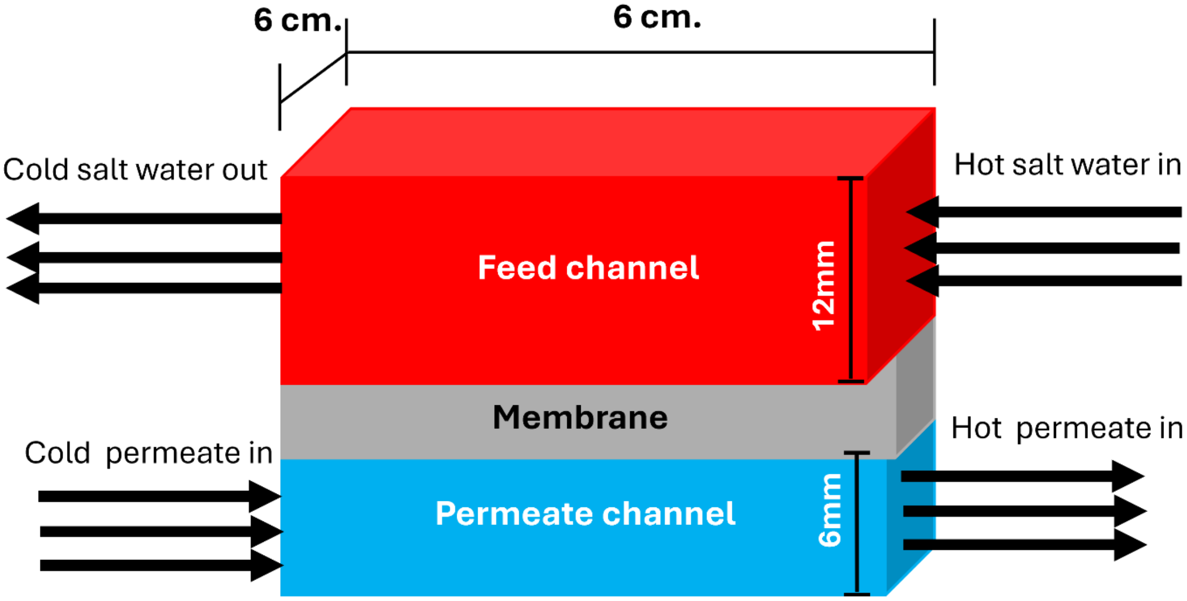

Figure S3. Schematic diagram of the 3D model flow directions

- A halogen lamp (J189 240V 1000W) was used for solar power generation, which was monitored by a solar meter (Evomex TM 750). An electronic analytical balance (sartorius CPA3245, accuracy 0.1 mg) was used to monitor water production in real-time, and Infrared (IR) images were taken with an infrared thermal imaging camera (FLIR IR lens f=0.65mm).The temperature profile of the prepared membranes was estimated using live streaming with the FLIR camera and its related software. The halogen lamps were on for 30-minute time intervals on the membrane surface, and then the surface temperature was captured and compared for the different prepared membranes.

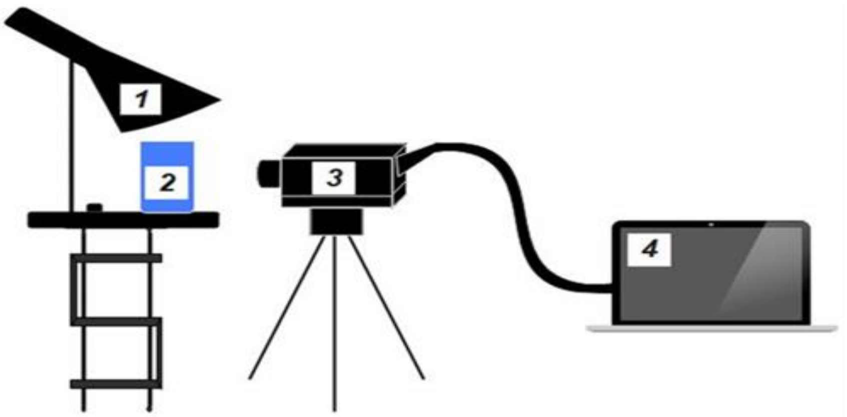

Figure S4. Photothermal evaluation experimental system set-up

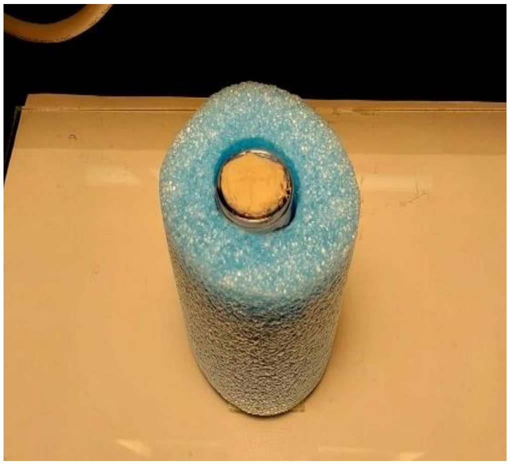

Figure S5. The experimental setup for water evaporation rate testing

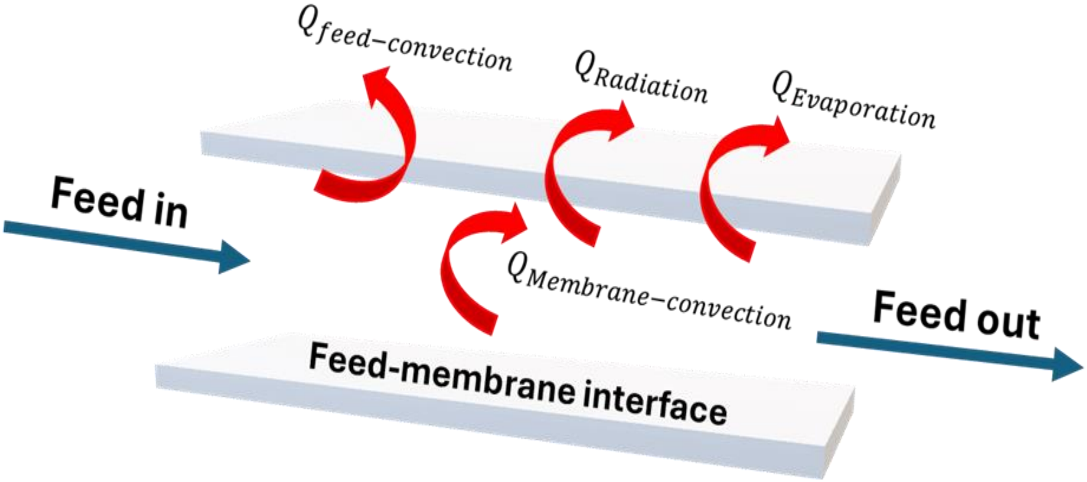

Figure S6. Heat fluxes of the adopted photothermal membrane distillation model

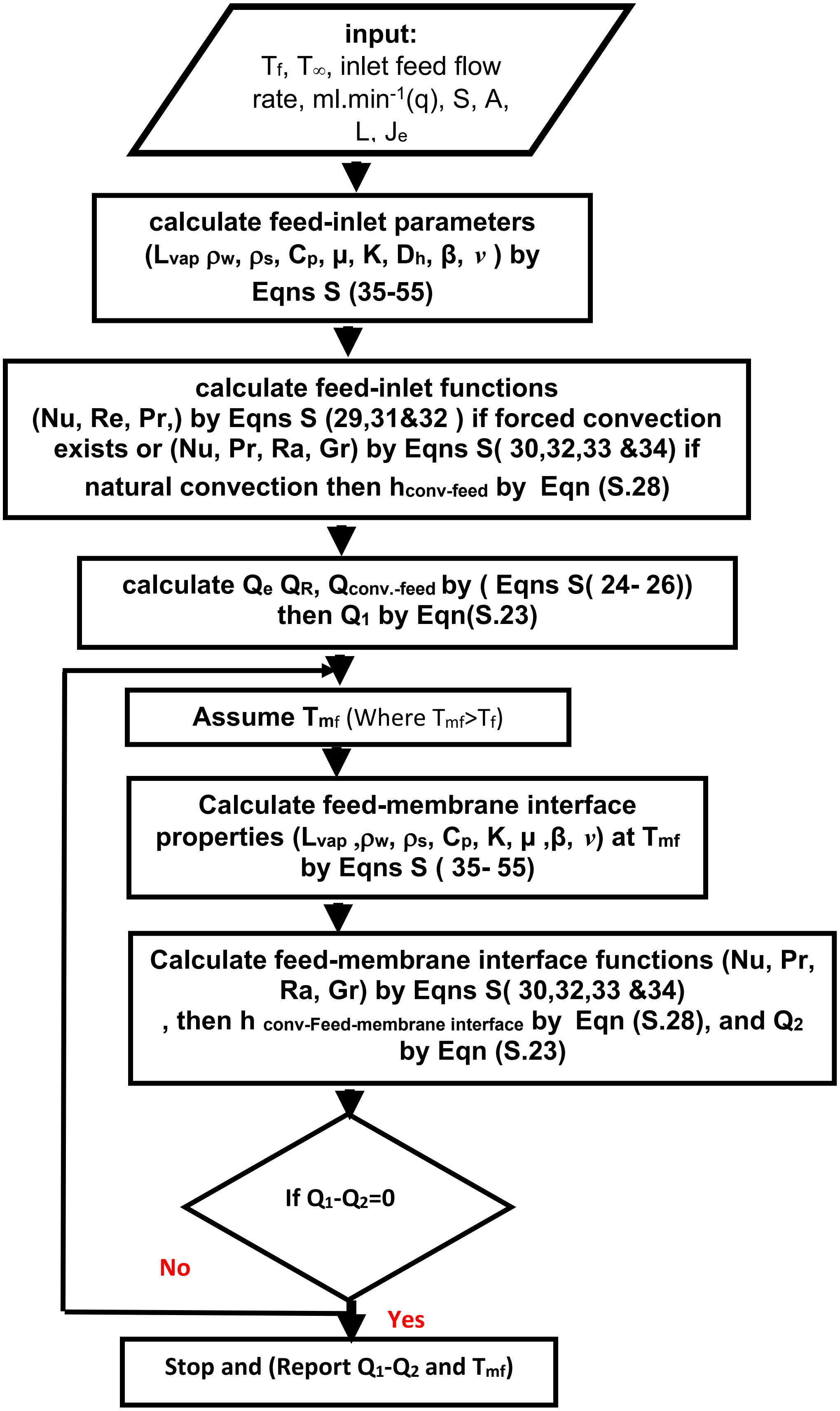

Figure S7. Flow chart of the numerical model

Section 2 Supplemental calculations

2.1. Porosity calculation

It was attained by a wet-dry gravimetric approach where each membrane was immersed in isopropanol (Alpha chemical, >99%) for 24 hrs., weighed after excess alcohol was removed (Ww), and then dried at 40 °C for 24 hrs. and reweighed (WD). The surface porosity of each membrane was calculated using **Eq. (S.1)**, which considers the weights of dry and wet specimens (WD and WW, respectively), the density of isopropanol at ambient temperature (ρIPA), the cross-section of the tested specimen (A), and its thickness (L) [1].

$$\%porosity = ((Ww - WD)\ /\rho IPA * A * L) * 100 \qquad \textbf{Eq. (S.1)}$$

2.2. Response surface calculations

The number of experiments (N) is defined by **Eq. (S.2)** [2].

$$\mathrm{N} = \mathrm{K}^2 + \mathrm{K} + \mathrm{C_P} \qquad \textbf{Eq. (S. 2)}$$

where K represents the number of factors and Cp the points at the center of the design, respectively (K= 3, Cp= 5). Using this formula, the number of experiments is calculated to be 17 runs. Additionally, the coded levels can be determined using **Eq. (S.3)**:

$$Z = \frac{Z_0 - Z_c}{\Delta Z} \qquad \textbf{Eq. (S.3)}$$

where: Z and Z0 are the coded and real levels of independent variables, respectively. ΔZ represents the step change, while Zc indicates the actual value at the central point.

2.3. Numerical model of direct contact membrane distillation

Numerical modelling was performed to predict the performance of the prepared membranes. The three-dimensional approach adopted by Mohammed Rabie and coauthors [3] and Ansys software (2020 R_2) was used. The Assumptions made in the current study include:

- Constant, laminar flow in both channels.
- No heat loss to the environment.
- Mass transfer occurs via molecular diffusion and Knudsen diffusion mechanisms.
- Membrane thermal conductivity is temperature-independent.
- No chemical reactions take place.

The governing equations for the MD unit are represented by the **Eqs. (S.4-6)** under these assumptions. The mass and heat transfer equations are expressed as follows:
- Continuity equation:

$$\nabla . U^{\rightarrow} = \frac{1}{\rho} . S_m \qquad \textbf{Eq. (S.4)}$$

- Momentum equation in both x and y directions:

$$(U^{\rightarrow} . \nabla) U^{\rightarrow} = -\frac{1}{\rho} \nabla P^{\rightarrow} + \vartheta \nabla^2 U^{\rightarrow} \qquad \textbf{Eq. (S.5)}$$

- Energy equation:

$$U^{\rightarrow} . \nabla T = \alpha \nabla^2 T + \frac{1}{\rho} S_e \qquad \textbf{Eq. (S.6)}$$

$U^{\rightarrow}$and $p^{\rightarrow}$ represent the velocity vector [$m.s^{-1}$] and pressure vector [Pa], respectively. While as ρ, ϑ, T, and α represent the water density [$kg.m^{-3}$], kinematic viscosity [$m^2.s^{-1}$], local temperature [K], thermal diffusivity [$m^2.s^{-1}$], Mass and heat sources are represented by S_m [$kg.m^{-3}. s^{-1}$] and S_e [$W.m^{-3}$], respectively. The continuity equation is amended to include the mass source, representing the quantity of permeate flux generated. This flux is extracted from the feed fluid and incorporated into the permeate fluid. Therefore, it has a negative value for the input channel and a positive value for the permeate channel. The heat source element is incorporated into the energy equation to denote the quantity of heat that is transferred from the feed channel to the permeate channel. Consequently, it bears the same signs as the mass source.

The MD model applied was using the commercially widely used PVDF membrane before being used in the investigation of the used manufactured membranes. For validation, the MD permeate mass flux, **Eq.(S.7)**, has been used as a system metric for validation defined as the quantity of vapor that flows through the membrane per unit area per unit time [4], [5].

$$J = C_m (P_f{}^v - P_p{}^v) \qquad \textbf{Eq. (S.7)}$$

The permeate mass flux transferred through the membrane and the mass transfer coefficient are represented by J [$kg.m^{-2}. s^{-1}$] and Cm [$kg.m^{-2}.s^{-1}.Pa^{-1}$], respectively. $P_f{}^v$ and $P_p{}^v$ represent the vapor pressure on the feed and permeate sides of the membrane, respectively. The vapor pressure at temperature (T_i)can be determined either by Antoine equation alone (for pure component) or with coupling with another equation for saline solution and for seawater as described by **Eqs. (S.8 & 9)** [6]–[8].

$$P_i{}^v = \exp\left(23.238 - \frac{3841}{T_i - 45}\right) \qquad \textbf{Eq. (S.8)}$$

$$P_{sw} = \frac{P}{1 + 0.57357\left(\frac{S}{1000 - S}\right)} \qquad \textbf{Eq. (S.9)}$$

Cm, the mass transfer coefficient, is determined by **Eqs. (S.10 & 11)** [4], [8].

$$C_m = [\frac{3}{2}\frac{\tau\delta}{\varepsilon r}(\frac{\pi RT}{8M})^{0.5} + \frac{\tau\delta}{\varepsilon}\frac{P_a}{PD}\frac{RT}{M}]^{-1}$$ **Eq. (S.10)**

$$P_D = 1.8958 * 10^{-5}T^{2.072}$$ **Eq. (S.11)**

The variables in the equations are defined as follows: δ represents the membrane thickness in meters, ε represents the porosity, τ represents the tortuosity, and r represents the average pore radius in meters. R is the universal gas constant with a value of 8.314 $J.mol^{-1}. K^{-1}$. T represents the mean membrane temperature. M represents the molecular weight of water, which is 0.018 $kg.mol^{-1}$. Pa represents the partial air pressure in the membrane pores in Pascals, P represents the total pressure in Pascals, and D represents the water diffusion coefficient in square meters per second. The membrane's tortuosity(τ)can be determined in terms of porosity (ε) using **Eq. (S.12)** [8].

$$\tau = \frac{(2-\varepsilon)^2}{\varepsilon}$$ **Eq. (S.12)**

Salt rejection is a crucial indicator of the produced water quality and is quantified using the formula given in **Eq. (S.13)** [9].

$$\text{Salt rejection} = 1 - \frac{\frac{m_{p2}s_{p2} - m_{p1}S_{p1}}{m_{p2}m_{p1}}}{s_{f1}}$$ **Eq. (S.13)**

where mp1 and mp2 are the weight of the distillate tank before and after the DCMD and SP1 and SP2 are the salinities (in ppm) of the initial and final permeate water in the permeate tank while Sf1 is the inlet feed salinity.

Another important factor is the temperature polarization coefficient (TPC), which quantifies the extent to which the temperatures of the membrane surfaces differ from the bulk temperature of the fluids. The expression for TPC is given in **Eq. (S.14)** [4].

$$TPC = \frac{T_{mf} - T_{mp}}{T_f - T_p}$$ **Eq. (S.14)**

where Tf and Tp are the bulk temperatures of feed and permeate fluids, respectively.

An additional crucial measure is the thermal efficiency of the MD system(η_{MD}), which quantifies the extent to which the available heat is effectively utilized for the evaporation of water and the production of the permeate flux and it is calculated using **Eq. (S.15)** [4].

$$\eta_{MD} = \frac{Q_v}{Q_v + Q_c} = \frac{J\Delta H}{J\Delta H + K_m\frac{(T_{mf} - T_{mp})}{\delta}}$$ **Eq. (S.15)**

The variable Qv $[W.m^{-2}]$ represents the rate of evaporative heat transfer, while Qc $[W.m^{-2}]$ represents the rate of conduction heat transfer through the membrane. These quantities are evaluated using **Eqs.(S.16-19)** [3].

$$Q_v = J\Delta H$$ **Eq. (S.16)**

$$\Delta H = [1.7535T_{mf} + 2024.3] * 1000$$ **Eq. (S.17)**

$$Q_c = K_m \frac{(T_{mf} - T_{mp})}{\delta}$$ **Eq. (S.18)**

$$K_m = \left[\left(\frac{\varepsilon}{K_{gas}} \right) + \left(\frac{1-\varepsilon}{K_s} \right) \right]^{-1}$$ **Eq. (S.19)**

J, ΔH, and T_{mf} are the permeate flux, the latent heat of vaporization [$J.kg^{-1}$], and the membrane temperatures at the feed side. respectively[10] and K_m is the membrane's overall thermal conductivity [$W.m^{-1}.K^{-1}$] [8].

The last system performance metric in this investigation is the specific energy consumption (SEC), $KWh.m^{-3}$, which assesses the energy required to generate 1 cubic meter of clean water and it is calculated using **Eq. (S.20)** [3].

$$SEC = \frac{Q_s}{\frac{JA}{\rho}} = \frac{\dot{m} C_P \triangle T}{\frac{C_m (P_f - P_p) A}{\rho}}$$ **Eq. (S.20)**

The variables Qs and A represent the heat provided to the entire process and the active area of the membrane, respectively. The variables $\dot{m}$, Cp, and ΔT define the feed mass flow rate, feed specific heat, and feed temperature increment, respectively.

2.4. Photothermal Activity evaluation

The obtained membranes' photothermal performance was evaluated using their temperature profile and water evaporation rate, which is estimated using the same system set up in **Fig (S.4)**. The water evaporation rate experiments were carried out using a self-made photothermal evaporation system. The system is close to the one used by (Xu and his team, 2020) [11].the photothermal composite membrane was pleated onto sponge hydrophilic foam and placed in a 30ml glass container containing distilled water. The beaker is placed in insulated casing foam, as shown in **Fig. (S.5)**. The sponge foam is connected to a thin rope from the same material immersed till the end of the beaker to transfer water to the membrane surface by the capillary action. The mass change of the system with different membranes was recorded over 30 min with an electronic balance. The weight of the evaluating system is recorded, from which the evaporation rate(E_m), $kg.m^{-2}hr^{-1}$., is calculated using **Eq. (S.21)** [12].

$$E_m = \frac{\Delta m}{A * t}$$ **Eq. (S.21)**

Where Δm, A, and t are the mass change due to water evaporation(kg.), the surface area of evaporation(m^2), and evaporation time (hr.), respectively.

The photothermal conversion efficiency($\eta_{solar-thermal}$) is evaluated using the subsequent **Eq. (S.22)** [12]:

$$\eta_{Solar-thermal} = \frac{E_m(C_p\Delta T + h_{lv})}{3600P_I} \quad \textbf{Eq. (S.22)}$$

In this equation, E_m represents the steam generation rate (kg.m^{-2}.hr.$^{-1}$) under solar irradiation, Cp is the specific heat capacity of water (a constant of 4.18 kJ. kg^{-1}. K^{-1}), ΔT is the temperature increase of the membrane surface under illumination, h_{lv} represents the real evaporation enthalpy ($h_{lv} = h_0/m_r$), h_0 is the evaporation enthalpy of water at the obtained temperature after irradiation, m_r is the mass change ratio in the presence and absence of the membrane and P_I is the power density of the incident light.

2.5. Photothermal membrane distillation evaluation

The photothermal membrane distillation (PMD) system performance was evaluated analytically and validated using the literature data and experimentally by the commercial PVDF membrane on the same system in **Fig (S.2)** using the halogen lamp as solar simulator source for feed heating to show the effectiveness of using solar power on the resultant permeate flux. The analytical model, based on the method described by Deoukchen Ghim et al., predicts the temperature at the feed-membrane interface. The model's main postulation, as shown in **Fig. (S.6)**, is that at equilibrium, the transfer of energy from the bulk water to its surroundings through heat convection, radiation, and evaporation is equal to the energy release from the membrane surface to the bulk water through heat convection [13].

The model steps are summarized in detail through the following flowchart, **Fig. (S.7),** and the following numerical equations:

-First, the heat fluxes equation is applied using **Eq. (S.23)** [13].

$$Q_1 = Q_e + Q_R + Q_{conv.-feed} = Q_2 = Q_{conv.-membrane\,surface} \quad \textbf{Eq. (S.23)}$$

Where, Q_e,Q_R, $Q_{conv.\text{-}feed}$, $Q_{conv.\text{-}membrane\ surface}$ (W.m^{-2}), are the heat fluxes from the feed side to the surrounding by evaporation, radiation, and convection respectively and, $Q_{conv.\text{-}membrane\ surface}$ is the heat dissipation from the feed-membrane interface to the bulk feed water by convection. They are calculated using **Eqs. (S.24)** through **(S.27).**

$$Q_e = J_e * \Delta L_{vap.} \quad \textbf{Eq. (S.24)}$$

Where the Je (kg.m^{-2}.hr^{-1}) and $\Delta L_{vap.}$ (kJ. kg^{-1}) are the water evaporation rate and the latent heat of vaporization at the bulk feed temperature.

$$Q_R = \varepsilon\sigma({T_f}^4 - {T_\infty}^4) \quad \textbf{Eq. (S.25)}$$

Where the ε is the water emissivity and σ=5.67037*10^{-08} (W.m^{-2}.K^{-4}) is the StefanBoltzmann constant, T_f and T_∞ are the bulk and surrounding temperatures respectively.

$$Q_{conv.-feed} = h_{conv.-feed} * (T_f - T_\infty) \quad \textbf{Eq. (S.26)}$$

$$Q_{conv.-membrane\,surface} = h_{conv.-membrane\,surface} * (T_{mf} - T_f) \quad \textbf{Eq. (S.27)}$$

And the heat transfer coefficient, h_{conv}(W.m^{-2}.K^{-1}) is calculated using **Eq. (S.28).**

$$h_{conv.} = \frac{N_u * K}{L}$$ **Eq. (S.28)**

Where K (W.m^{-1}. °C^{-1}) is the solution thermal conductivity and L(m) is the membrane characteristic length (Area/perimeter) and the Nu, Nusselt number could be calculated using **Eqs. S (29 & 30)** according to the convection type.

For **Forced-convection** [10]: $N_u = 0.13 * R_e^{0.64} * P_r^{0.38}$ **Eq. (S.29)**

For **Natural-convection**[13]: $N_u = 0.134 * R_a^{0.34}$ **Eq. (S.30)**

Where: Re, Pr, Ra and Gr are dimensionless parameters calculated using **Eqs. S (31)** through **(55)**.

- $R_e = \frac{\rho_s V D_h}{\mu}$ [4] **Eq. (S.31)**
- $P_r = \frac{\mu C_p}{K}$ [13] **Eq. (S.32)**
- $R_a = G_r * P_r$ [13] **Eq. (S.33)**
- $G_r = \frac{g\beta(T_{mf}-T_f)L^3}{v^2}$ [13] **Eq. (S.34)**

- $\Delta L_{vap} = 2501.897149 - (2.407064037 * T) + (1.192217 * 10^{-3} * T^2) - (1.5863 * 10^{-5} T^3)$ **Eq. (S.35)**

- $\rho_w = 999.842594 + (6.893952 * 10^{-2} T) - (9.095290 * 10^{-3} T^2) + (1.001685 * 10^{-4} T^3) + (1.120083 * 10^{-6} T^4) + (6.536336 * 10^{-9} T^5)$ **Eq. (S.36)**

- $\rho_s = \rho_w + A_s S_A + B_s S_A^{1.5} + (5.281399 * 10^{-4}) S_A^2$ **Eq. (S.37)**

- $S_A = \frac{35.16054}{35} S$ **Eq. (S.38)**

- $A_S = (8.246111 * 10^{-1}) - (3.956103 * 10^{-3} T) + (7.274549 * 10^{-5} T^2) - (8.239634 * 10^{-7} T^3) + (5.332909 * 10^{-9} * T^4)$ **Eq. (S.39)**
- $B_s = (-6.006733 * 10^{-3}) + (7.970908 * 10^{-8} * T) - (1.018797 * 10^{-6} T^2)$ **Eq. (S.40)**

- $\mu = \mu_w \mu_c * 10^{-3}$ **Eq. (S.41)**
  - $\ln(\mu_w) = -3.79418 * \frac{604.129}{139.18 + T}$ **Eq. (S.42)**
  - $\mu_c = 1 + A_\mu S + B_\mu S^2$ **Eq. (S.43)**
  - $A_\mu = 1.474 * 10^{-3} + (1.5 * 10^{-6} T) - (3.927 * 10^{-8} T^2)$ **Eq. (S.44)**
  - $B_\mu = 1.0734 * 10^{-5} - (8.5 * 10^{-8} T) + (2.23 * 10^{-10} T^2)$ **Eq. (S.45)**
- $C_p = (A_c + B_c T + C_c T^2 + D_c T^3) * 10^{-3}$ **Eq. (S.46)**
  - $A_c = 4206.8 - 6.6197S + (1.2288 * 10^{-2} S^2)$ **Eq. (S.47)**
  - $B_c = -1.1262 + (5.4178 * 10^{-2} S) - (2.2729 * 10^{-4} S^2)$ **Eq. (S.48)**
  - $C_c = (1.2026 * 10^{-2}) - (5.3566 * 10^{-4} S) + (1.8906 * 10^{-6} S^2)$ **Eq. (S.49)**

- $D_c = (6.8777 * 10^{-7}) + (1.517 * 10^{-6}S) - (4.4268 * 10^{-9}S^2)$ **Eq. (S.50)**

- $\log_{10} K = A_k + 0.434 * \left(2.3 - \frac{343.5+(3.7*10^{-2})S}{T+273.15}\right)$ **Eq. (S.51)**

- $A_k = \log_{10} 240 + (2 * 10^{-4}\, S)$ **Eq. (S.52)**

- $D_h = \frac{4Area}{perimeter}$ **Eq. (S.53)**

- $\beta = -\frac{1}{\rho}(\frac{\partial \rho_s}{\partial T})_p$ **Eq. (S.54)**

- $\nu = \frac{\mu}{\rho_s}$ **Eq. (S.55)**

To estimate the optimal T_{mf} for the model, two Excel functions were used namely, Excel's Goal Seek function and Excel's Solver. With Excel's Goal Seek function, the T_{mf} is iteratively adjusted until the (Q_1-$Q_2 \simeq 0$). Following this, Excel's Solver was applied by optimizing the T_{mf} through adjusting the (Q_1-Q_2) to be equal to zero, within the constraints of $Q_1 = Q_2$ and $T_{mf} \geq T_f$. After the Solver's iterative process converged, the optimum T_{mf} fulfilled the constraints for further permeate flux measurements.

The photothermal flux was then calculated using the same equations of the DCMD, **Eqs S (7) through (12)** for the commercial PVDF membrane, and the result was validated with the experimental setup, **Fig. (S.2)**, and further with the literature results. After validation, the model was used for the evaluation of the PS-prepared membranes (whether plain or composites) to have insights into their PMD performance. Finally, the conventional DCMD flux is determined by the Ansys software under the same process conditions in the absence of solar irradiation to show the effect of solar power on water productivity.

2.6. Uncertainty Measurements

For fiber Diameter and pore size measurements

It is determined using Image J software processing of the SEM images with a precision of 3 decimal places at min 20 places. By using the following equations [14]:

- **The mean (average) calculation:**

$$\bar{x} = \frac{1}{n}\sum_{i=1}^{n} x_i$$

where x_i are the individual measurements and n is the total number of measurements.

- **Standard Deviation (SD) calculation, which estimates the variability in your measurements:**

$$SD = \sqrt{\frac{1}{n-1}\sum_{i=1}^{n}(x_i - \bar{x})^2}$$

- **Standard Error of the Mean (SEM) to estimate the precision of the mean:**

$$\mathrm{SEM} = \frac{\mathrm{SD}}{\sqrt{\mathrm{n}}}$$

- **Report the measurement as:**

$$\bar{\mathrm{x}} \pm \mathrm{SEM}$$

Supplementary references

[1] Mohsenpour, S.; Safekordi, A.; Tavakolmoghadam, M.; Rekabdar, F.; Hemmati,M. Comparison of the membrane morphology based on the phase diagram using PVP as an organic additive and TiO2 as an inorganic additive. Polymer 2016, 97, 559–568. https://doi.org/10.1016/j.polymer.2016.05.069.

[2] Gorji, M.; Sadeghian Maryan, A. Breathable-windproof membrane via simultaneous electrospinning of PU and P(AMPS-GO) hybrid nanofiber: Modeling and optimization with response surface methodology. Journal of Industrial Textiles 2018, 47, 1645–1663. https://doi.org/10.1177/1528083717705622.

[3] Rabie, M.; Ali, A.Y.; Abo-Zahhad, E.M.; Elqady, H.I.; Elkady, M.; Ookawara, S.; El-Shazly, A.; Salem, M.S.; Radwan, A. Thermal analysis of a hybrid high concentrator photovoltaic/membrane distillation system for isolated coastal regions. Sol. Energy 2021, 215, 220–239. https://doi.org/10.1016/j.solener.2020.12.029

[4] Qtaishat, M.; Matsuura, T.; Kruczek, B.; Khayet, M. Heat and mass transfer analysis in direct contact membrane distillation. Desalination 2008, 219, 272–292. https://doi.org/10.1016/j.desal.2007.05.019.

[5] Rochd, S.; Zerradi, H.; Mizani, S.; Dezairi, A.; Ouaskit, S. Modelisation of Membrane Distillation: Mass and Heat Transfer in Air Gap Membrane Distillation. J. Membr. Sci. Technol. 2016, 6, 1–9. https://doi.org/10.4172/2155-9589.1000154.

[6] Phattaranawik, J.; Jiraratananon, R.; Fane, A. G. Heat transport and membrane distillation coefficients in direct contact membrane distillation. J. Memb. Sci. 2003, 212, 177–193. https://doi.org/10.1016/S0376-7388(02)00498-2.

[7] Soukane, S.; Naceur, M. W.; Francis, L.; Alsaadi, A.; Ghaffour, N. Effect of feed flow pattern on the distribution of permeate fluxes in desalination by direct contact membrane. Distillation 2017, 418, 43–59. https://doi.org/10.1016/j.desal.2017.05.028.

[8] Lawal, D. U.; Khalifa, A. E. Flux Prediction in Direct Contact Membrane Distillation. Int. J. Mater. Mech. Manuf. 2014, 2, 302–308. https://doi.org/10.7763/IJMMM.2014.V2.147.

[9] Salem, M. S.; El-Shazly, A. H.; Nady, N.; Elmarghany, M. R.; Sabry, M. N. PES/PVDF blend membrane and its composite with graphene nanoplates: Preparation, characterization, and water desalination via membrane distillation. Desalin. Water Treat. 2019, 166, 9–23. https://doi.org/10.5004/dwt.2019.24611.

[10] Bahmanyar, A.; Asghari, M. Khoobi, N. Numerical simulation and theoretical study on

simultaneously effects of operating parameters in direct contact membrane distillation. Chem. Eng. Process. Process Intensif. 2012, 61, 42–50. https://doi.org/10.1016/j.cep.2012.06.012.

[11] Xu, Y.; Ma, J.; Han, Y.; Xu, H.; Wang, Y.; Qi, D.; Wang, W. A simple and universal strategy to deposit Ag/polypyrrole on various substrates for enhanced interfacial solar evaporation and antibacterial activity. Chem. Eng. J. 2020, 384, 123379. https://doi.org/10.1016/j.cej.2019.123379.

[12] Fang, K.; Du, C.; Zhang, J.; Zhou, C.; Yang, S. Molecular engineering of a synergistic photocatalytic and photothermal membrane for highly efficient and durable solar water purification. J. Memb. Sci. 2022, 663, 121037. https://doi.org/10.1016/j.memsci.2022.121037.

[13] Ghim, D.; Wu, X.; Suazo, M.; Jun, Y. Achieving maximum recovery of latent heat in photothermally driven multi-layer stacked membrane distillation. Nano Energy 2020, 80, 105444. https://doi.org/10.1016/j.nanoen.2020.105444.

[14] Montgomery, D. C., Runger, G. C. (2011). Applied statistics and probability for engineers, 5th ed.; John Wiley & Sons, Inc.: Hoboken, New Jersey, USA, 2011; ISBN 978-0-470-05304-1
